# How can we make valid and useful comparisons of different health care systems?

**DOI:** 10.1111/1475-6773.13883

**Published:** 2021-11-10

**Authors:** Andrew Street, Peter Smith

**Affiliations:** ^1^ Department of Health Policy London School of Economics and Political Science London UK; ^2^ Centre for Health Economics University of York York UK

## 1 INTRODUCTION

It is important to understand and seek to reduce unwarranted variations in health treatments in order to improve health outcomes, inequalities in access and health system efficiency. Traditionally this monitoring function has been undertaken at national or subnational levels, as a means of identifying potential improvements in clinical practice and the performance of the health systems. However, international comparison of treatments is also recognized as being an important tool for assessing performance and prompting improvement, especially when examining whether the design of the health system needs reconsideration.^1^

However, making international comparisons is not straightforward, with two challenges standing out: first, the difficulty of making valid like‐for‐like comparisons; second, whether the analysis can help drive performance improvements.^2^

## 2 MAKING VALID COMPARISONS

In relation to the first challenge, to make international comparisons, a key requisite is that the data used for the analysis are measured accurately and consistently for all countries subject to the comparative exercise. If not, comparative differences may derive from differences in the data rather than being a reflection of relative performance. Perhaps the most important of international standards on data specification regarding health care expenditure is the System of Health Accounts, which apply the world over.^3^, ^4^ Among high‐income countries, the Organisation for Economic Co‐operation and Development (OECD) has a long running series of “Health Statistics” that document trends in the macro characteristics of health systems, such as total spending and length of hospital stay.^5^ In 2001, the OECD initiated a Health Care Quality Indicators (HCQI) project that compares quality and safety across high‐income countries.^6^ By 2019, the project had assembled a total of 61 indicators across 38 countries, covering the following “themes”: Primary care, prescribing, acute care, mental health care, cancer care, patient safety, and patient experiences. And The Commonwealth Fund regularly publishes its “Mirror Mirror” reports comparing the performance of the United States with that of 10 other high‐income countries.^7^

Notwithstanding efforts to construct these datasets, they come with significant “health warnings” about the data therein, with copious footnotes noting caveats for each variable. Even the definition of the “health system” varies across countries, for example, in the extent to which long‐term care is considered a part of the health system. Countries also employ different definitions of what constitutes a hospital bed, a doctor, or a nurse. Even the definition of a “patient” varies: some countries are able to track patients across institutions involved in delivering treatment and support along the care pathway; in other countries, it is very difficult to identify how patients access care in different settings. Similarly, the processes of care, such as hospital waiting times, or the outcomes of care, notably its impact on health status, are measured and reported differently, if at all. Inevitably, therefore, analyses employing inconsistently defined or inaccurately measured data may not be able to draw valid conclusions about comparative performance.

## 3 MAKING USEFUL COMPARISONS

Many studies that make international comparisons use highly aggregated data, giving rise to the second challenge: if analyses suggest poor performance, what specific action can be taken in response? Decision makers need to know where the problems lie. Is poor performance due to the health system alone or a reflection of society more generally and the social determinants of health, such as poverty, housing, and environmental conditions? If the health system, are there problems across social, primary, secondary, and tertiary care, or are some sectors performing poorly and others relatively well? Are problems evident for all health problems or mostly driven by how care is organized for particular conditions, such as maternity or cancer care? Analyses based on aggregate data offer no insights into such questions and, hence, no intelligence as to what action should be taken.

## 4 ADDRESSING THESE CHALLENGES

In fact, both challenges can be met fairly easily. All that is required is a more focused analysis. Instead of trying to analyze the health system as a whole or an entire sector within the system, a growing body of research assessing relative performance is highly focused, concentrating on how care is delivered for specific types of patients. The Dartmouth Atlas was an early leader in this endeavor, using routine data to examine variation in care across the United States.^8^, ^9^ Similar atlases of variation have been compiled by other countries, and the European Collaboration for Healthcare Optimization (ECHO) project applied the approach in making comparisons across European countries.^10^ Another example within Europe was the HealthBasket project, which sought to compare across nine countries the resources used and benefit packages for 10 common treatment “vignettes.”^11^

The key underlying principle of such research is to ensure that like‐for‐like comparisons of the same types of patients are being made, whether these types are defined using vignettes or patients are identified by means of precise specification of the diagnosis codes. This precision provides confidence that differences that emerge from these performance analyses are not due to differences in those being studied but to how they are being cared for. And by undertaking focused analyses of clearly defined sets of patients, the analyses direct attention: if poor performance is observed, care for these specific patients needs to be reviewed.

## 5 INSIGHTS FROM THE ICCONIC PROJECT

The approach to the research reported in this special issue is consistent with this body of literature. The papers examine the characteristics and health care utilization and outcomes across 11 countries for people with high need and high costs (HNHC). The first paper^12^ sets out the methodological approach, notably the justification for focusing on two particular types of HNHC patients: older frail adults with a hip fracture and older people with complex multimorbidity including heart failure and diabetes. These are important high needs “personas,” as they are highly prevalent, and treatment is costly and delivered across multiple care settings. In each country, individual‐level data about people who matched these personas were extracted from routine datasets and linked across seven settings: hospital care, primary care, outpatient care, rehabilitation, long‐term care, home care, and pharmaceuticals. The paper by Figueroa et al.^13^ examines variations in health care utilization and spending within care settings and across countries for patients with heart failure and diabetes. This involves constructing and comparing Lorenz curves to assess whether the distributions of service use and utilization are similar, which they tend to be.

The next two papers describe patterns of resource utilization and expenditure for heart failure and diabetic patients^14^ and hip fracture patients,^15^ respectively. For both personas, patients were identified when first hospitalized, and their use of services was calculated for the year prior to hospitalization and for the year immediately afterwards. Comparison of these utilization and expenditure profiles across countries yielded interesting insights. The United States stood out as having the highest expenditure, driven by a combination of three factors. First, input prices are higher. Second, patients use more services, notably having longer hospital stays and more rehabilitation than elsewhere. Third, there are different delivery patterns across settings, with patients in the United States more likely than in other countries to receive follow‐up care in more expensive specialist clinics than in cheaper primary care settings. The first of these drivers has long been known^16^ but, by linking data across settings, this research has been able to offer novel evidence about the other two drivers.

The question then arises: Is there any relationship between resource use and patient outcomes? This is examined by Papanicolas et al.,^17^ who assess readmission rates and mortality rates for these patients. Both outcomes are worse for those with heart failure and diabetes than for those suffering a hip fracture. But there are cross‐country differences as well. Mortality rates for both personas are worse in England than elsewhere, which might partly reflect the spending in that country. But that is too simple an explanation: after England, mortality rates are highest in the United States, implying that there is no obvious return from the higher spending there.

The paper by Papanicolas et al.^18^ provides a greater analysis of the care provided to hip fracture patients for those who survived for at least 1 year following their hospitalization. Post‐acute expenditure is lower for countries, notably Germany, that substitute more expensive institutional rehabilitative care for relatively cheaper home‐based care.

The final paper by Blankart et al.^19^ provides a more in‐depth analysis of service use and spending in the last 12 months of life for those hip fracture patients who died. In common with the rest of the literature on the subject,^20^ the paper demonstrates that utilization and costs increase as people near their deaths, given their receipt of end‐of‐life care (EoLC). But the paper also reveals how EoLC varies across countries. Most strikingly, the likelihood of dying in a hospital rather than at home or in a hospice is lowest in Australia and New Zealand and highest in England and Spain. The paper also shows that EoLC spending is higher in Canada and the United States, driven more by higher prices than greater use of services.

## 6 MOVING FORWARD

Taken together, the papers in this special issue represent important advances in international comparison of treatments and outcomes. They have demonstrated that, notwithstanding major differences in data specification and collection mechanisms, routine administrative data can act as a powerful basis for comparison between health systems in high‐income countries, yielding novel insights. While there are few formal international data standards, there are sufficient commonalities among information systems in the selected countries to make meaningful comparisons of how identical patients are treated in different countries.

The emphasis on specifying “personas” of specific patient types obviates the need for complex risk adjustment mechanisms, which often compromises confidence in comparisons of more heterogeneous treatment groups. The drawback of using such personas is their relatively narrow focus. However, the personas used in these studies are for high prevalence and high cost patients for whom a range of possible treatment pathways exist. Findings for these groups will in themselves be directly relevant for a significant proportion of health system spending, and are also likely to be indirectly relevant to a wide range of other high needs patients.

The challenges associated with identifying comparable personas across countries should not be underestimated. There remain differences in the use of the International Classification of Diseases, and there are variations in the extent to which comorbidities are recorded. Linkage of patient pathways across health care providers is highly variable, and there is little standardization of procedure codes and resource utilization metrics. So far as is feasible, the methodology used in these studies successfully exploits commonalities between countries and highlights where comparisons remain unreliable or impossible.

Perhaps the biggest weakness identified in these studies was the shortage of useful measures of patient outcome, relying primarily on examining differences in mortality. There remain few international standards regarding patient‐reported outcomes^21^ or process measures such as waiting times.^22^ Such metrics are becoming increasingly important indicators of health care quality, and a failure to consider them leads to an incomplete picture of health system performance. There is a clear need for the development of widely accepted quality metrics that can be used for clinical management as well as comparisons within and across health systems.

Of course, the ultimate touchstone for the success of initiatives such as the ICCONIC project is the extent to which they promote real change in health systems and data collection. The researchers and funders will need to work hard to ensure that the key messages from these published papers reach audiences who can take appropriate action to better support health care users with high needs and high costs.
